# Early detection of emerging SARS-CoV-2 variants of interest for experimental evaluation

**DOI:** 10.3389/fbinf.2022.1020189

**Published:** 2022-10-24

**Authors:** Zachary S. Wallace, James Davis, Anna Maria Niewiadomska, Robert D. Olson, Maulik Shukla, Rick Stevens, Yun Zhang, Christian M. Zmasek, Richard H. Scheuermann

**Affiliations:** ^1^ Department of Informatics, J. Craig Venter Institute, La Jolla, CA, United States; ^2^ Department of Computer Science and Engineering, University of California, San Diego, San Diego, CA, United States; ^3^ Division of Data Science and Learning, Argonne National Laboratory, Lemont, IL, United States; ^4^ University of Chicago Consortium for Advanced Science and Engineering, University of Chicago, Chicago, IL, United States; ^5^ Computing Environment and Life Sciences, Argonne National Laboratory, Argonne, IL, United States; ^6^ Department of Computer Science, University of Chicago, Chicago, IL, United States; ^7^ Department of Pathology, University of California, San Diego, San Diego, CA, United States; ^8^ Division of Vaccine Discovery, La Jolla Institute for Immunology, La Jolla, CA, United States; ^9^ Global Virus Network, Baltimore, MD, United States

**Keywords:** SARS-CoV-2, variants of concern, omicron, delta, viral evolution, genomic surveillance, bioinformatics, pandemic response

## Abstract

Since the beginning of the COVID-19 pandemic, SARS-CoV-2 has demonstrated its ability to rapidly and continuously evolve, leading to the emergence of thousands of different sequence variants, many with distinctive phenotypic properties. Fortunately, the broad application of next generation sequencing (NGS) across the globe has produced a wealth of SARS-CoV-2 genome sequences, offering a comprehensive picture of how this virus is evolving so that accurate diagnostics, reliable therapeutics, and prophylactic vaccines against COVID-19 can be developed and maintained. The millions of SARS-CoV-2 sequences deposited into genomic sequencing databases, including GenBank, BV-BRC, and GISAID, are annotated with the dates and geographic locations of sample collection, and can be aligned to and compared with the Wuhan-Hu-1 reference genome to extract their constellation of nucleotide and amino acid substitutions. By aggregating these data into concise datasets, the spread of variants through space and time can be assessed. Variant tracking efforts have initially focused on the Spike protein due to its critical role in viral tropism and antibody neutralization. To identify emerging variants of concern as early as possible, we developed a computational pipeline to process the genomic data and assign risk scores based on both epidemiological and functional parameters. Epidemiological dynamics are used to identify variants exhibiting substantial growth over time and spread across geographical regions. Experimental data that quantify Spike protein regions targeted by adaptive immunity and critical for other virus characteristics are used to predict variants with consequential immunogenic and pathogenic impacts. The growth assessment and functional impact scores are combined to produce a *Composite Score* for any set of Spike substitutions detected. With this systematic method to routinely score and rank emerging variants, we have established an approach to identify threatening variants early and prioritize them for experimental evaluation.

## Introduction

The ongoing evolution of SARS-CoV-2 has remained a persistent public heath challenge throughout the entire course of the COVID pandemic. Since the first strain of the virus was isolated and fully sequenced (Wu et al., 2020), SARS-CoV-2 has evolved into thousands of lineages and sub-lineages containing unique combinations of mutations, also known as variant constellations, with many of these mutations leading to altered virus phenotypes, in terms of antigenicity, transmissibility, and viral fitness (Harvey et al., 2021; Derek et al., 2022). In order to characterize and label the rapidly growing collection of variants, the scientific community has relied on the PANGO Lineage (Rambaut et al., 2020) nomenclature and WHO classifications as naming schemes for these variants. The continual emergence of SARS-CoV-2 variants has caused public health agencies to categorize selected lineages depending on their predicted importance. In the United States, the CDC has defined four categories of variants: Variants Being Monitored (VBM), Variants of Interest (VOI), Variants of Concern (VOC), and Variants of High Consequence (VOHC). Classification is dependent on viral growth dynamics and the level of threat to preexisting immunity or therapeutic efficacy. Notable Variants of Concern include B.1.1.7 (WHO class Alpha), B.1.617.2 (Delta), and the most recent B.1.1.529/BA.1-BA.5 (Omicron). (Centers for Disease Control and Prevention, 2019).

It has been a consistent pattern throughout the course of the pandemic for SARS-CoV-2 to acquire genetic changes of functional and epidemiological importance, beginning with the observation as early as spring 2020 that the Spike protein mutation, D614G, was associated with higher viral loads and was under positive selection (Korber et al., 2020). Since then, with the emergence of Alpha and Delta, Spike mutations such as N439R, N501Y, E484K, and P681H, have been linked to increased ACE2 binding affinity, antibody binding escape, and enhanced viral replication (Starr et al., 2020; Greaney et al., 2021a; Liu et al., 2021). The most recently emerged Variant of Concern, Omicron, has been reported to contain constellations of mutations across the n-terminal domain (NTD) and receptor binding domain (RBD). This has resulted in high levels of viral antigenic escape, and partial or complete resistance to the majority of available therapeutic monoclonal antibodies and, to a large extent, infection or vaccine-elicited polyclonal antibody binding and neutralization (Planas et al., 2022; Sievers et al., 2022). The astonishing ability of SARS-CoV-2 to rapidly evolve into variants with expanded cellular tropism, enhanced replication, increased transmissibility, and evasion of preexisting immunity has triggered the scientific community to band together and critically monitor the evolution of this virus through efforts like the US National Institute of Allergy and Infectious Diseases (NIAID)’s SARS-CoV-2 Assessment of Viral Evolution (SAVE) program (DeGrace et al., 2022) and COVID-19 Genomics UK Consortium (COG UK) (COVID-19 Genomics UK Consortium, 2019), which seek to iteratively provide a risk-assessment of emerging variants of interest and offer recommendations towards an optimal public health response.

A key approach to successfully monitor viral evolution, and thereby set the stage for risk assessment and mitigation, is through genomic surveillance (Deng et al., 2020; Korber et al., 2020). Thanks to the rapid deployment of next generation sequencing (NGS) laboratories, SARS-CoV-2 genome sequences from millions of infected individuals across the globe have been determined and deposited into public databases (Chiara et al., 2021) such as the National Center for Biotechnology Innovation (NCBI) GenBank (Sayers et al., 2022), the Global Initiative on Sharing Avian Influenza Data (GISAID) (Khare et al., 2021), and the Bacterial and Viral Bioinformatics Resource Center (BV-BRC) (BV-BRC, 2022). These data have been dynamically growing at a rapid pace since the early stages of the pandemic and have allowed researchers to pinpoint mutations under positive or negative selection through the evaluation of occurrence rates of synonymous *versus* non-synonymous nucleotide substitutions and the identification of the mutational drivers of evolution by modeling growth as linear combinations of the effects of individual mutations (Martin et al., 2021; Obermeyer et al., 2022). Overall, the amount of SARS-CoV-2 genomic sequencing being carried out on a global scale and the bioinformatics capabilities to understand these data has opened opportunities for diverse research into strategies for the early detection of emerging variants of concern based on epidemiological dynamics and predicted functional characteristics in near real time (DeGrace et al., 2022; Maher et al., 2022; Obermeyer et al., 2022). The concept of early detection is a key aspect of the NIAID SAVE program - to assess risk of novel emerging variants and select and prioritize variants for *in vitro* and *in vivo* experimentation (see DeGrace et al., 2022 for a complete description of the NIAID SAVE program for more details of the integrated workflow).

Here we present a computational heuristic developed for early detection of SARS-CoV-2 emerging variants of interest, which combines spatiotemporal sequence prevalence dynamics and geographic spread with functional impact prediction, that can be used to rank variants composed of constellations of substitutions. The methods described have been used to rank emerging variants of interest for the NIAID SAVE consortium Early Detection group every month since March 2021 to inform wet-lab experimentation.

## Methodology and results

### Algorithms for early detection

#### Sequence prevalence dynamics

The BV-BRC team has developed the SARS-CoV-2 Early Detection and Analysis Pipeline to offer informatics support for analyzing emerging SARS-CoV-2 variants from genomic sequencing data and associated epidemiological metadata processed from public databases on a recurring basis. The data used as input for this pipeline can be downloaded from either the NCBI GenBank (Sayers et al., 2022), Virus Pathogen Resource (ViPR) (Pickett et al., 2012), BV-BRC (BV-BRC, 2022) databases, or EpiCov portal of GISAID (Khare et al., 2021). A primary aspect of this pipeline is the ability to capture four measures of sequence prevalence dynamics for each unique constellation of SARS-CoV-2 genome sequence substitutions segregated by month and geographic location with a focus on substitutions that result in amino acid changes in the encoded proteins. The sequence prevalence dynamics include 1) the total genome sequence counts, 2) the genome sequence counts for specific lineages and variants, 3) the sequence prevalence of these lineages and variants, and 4) the growth rates of these lineages and variants from month to month (see Supplementary Material for more details). These sequence prevalence dynamics are calculated for individual PANGO lineages, single amino acid substitutions found in any SARS-CoV-2 protein, and unique combinations of protein substitutions denoted as “covariants”, which we refer to collectively as “variants”. (Note that each covariant also belongs to one or more specific PANGO lineages based on the nomenclature status of the original sequence; multiple covariants of a single protein can belong to the same PANGO lineage.) In this work, we focus on the analysis of Spike covariants since this has been the initial focus of the NIAID SAVE program, however the algorithm is also able to quantify the dynamics of covariants in non-spike proteins as well.

#### Capturing the dynamics of emerging variants

A customized pipeline is used to compute sequence prevalence dynamics upon capturing SARS-CoV-2 variants, either single amino acid substitutions, covariant combinations or PANGO lineage designations, and isolation metadata (geographic location and date of sample collection) from the genomic sequence databases. Strict quality control criteria are used to filter out genomes with high numbers of ambiguous or indeterminate base, low sequence length coverage, missing viral names, or improper metadata such as incorrect representation of dates or location names. Genomes passing quality control are then pairwise aligned to the Wuhan-Hu-1 reference genome (NC_045512.2), and the constellation of variants extracted for the Spike protein, or for the entire SARS-CoV-2 proteome (all 16 non-structural proteins as well as E, M, N, S, ORF3a, ORF6, ORF7a, ORF7b, ORF8, ORF9b, ORF10, and ORF14). Variant constellations are then partitioned into temporal period (month by default) and geographic region (country by default) groups. The total number of genomes isolated for the spatiotemporal groups is calculated and serves as the denominator for the sequence prevalence ratio (formula 1). Variant constellation counts and total isolate counts per region per time period are used to compute epidemiological dynamics, namely the prevalence and growth rates of variants between time periods as shown in formulas (1) and (2).

$$Prevalence\;Ratio=\frac{\#\;Variant\;Sequences\;in\;Region\;R\;during\;Period\;P}{\#\;Total\;Sequeces\;Isolated\;in\;Region\;R\;during\;Period\;P}$$ (1)

$$Growth\;Rate=\frac{Prevalence\;Ratio\;in\;Region\;R\;during\;Period\;P\;}{Prevalence\;Ratio\;in\;Region\;R\;during\;Period\;P-1}$$ (2)

Running this process to identify variant constellations and acquire region-period counts directly from the raw sequence data is computationally expensive for large batches of genomes and requires high-performance computing resources. An alternative approach is to use preprocessed data provided in GISAID metadata files, which include the Spike variant constellations, geographic location, date of collection, and basic quality control data such as the sequence length and ambiguous nucleotide content and can be downloaded with a registered GISAID account.

#### Sequence prevalence score

To identify SARS-CoV-2 variations with concerning epidemiological dynamics, a scoring heuristic based on sequence prevalence ratio (Formula 1), growth in sequence prevalence (Formula 2), and geographic spread was devised - the Sequence Prevalence Score. The method first segregates variants into geographic regions (e.g., country by default). Next, only variants with a sequence count greater than 10 in the most recent month are retained to control for large apparent growth rates associated with small numbers. Any variants that were filtered out are assigned a score of 0. To calculate the Sequence Prevalence Score, the most recent time periods (e.g., three months by default) of data are used. A score of 1 is assigned for every geographic region/time period combination in which the sequence prevalence is >5% or the growth rate from the previous month is > 5-fold by default. These numbers are then summed to obtain the final Sequence Prevalence Score across all geographic region/time period combinations. Table 1 shows a ranking using the Sequence Prevalence Score to prioritize variants from GISAID data processed in November 2021, which is considered the initial month of emergence of the Omicron variant. In this ranking, the Sequence Prevalence Score for the Omicron variant is still relatively small but detectable.

**TABLE 1:** Global Spike Covariant Rankings by the Sequence Prevalence Score. Ranking of covariants based on Sequence Prevalence Scores calculated from GISAID metadata up to November 2021 is shown. The initial emergence of the Omicron variant is being captured in this ranking, but at a relatively low rank.

WHO label	Covariant	Sequence prevalence score	Rank
Delta	T19R, G142D, E156G, F157-, R158-, L452R, T478K, D614G, P681R, D950N	123	1
Delta	T19R, T95I, G142D, E156G, F157-, R158-, L452R, T478K, D614G, P681R, D950N	92	2
Delta	T19R, E156G, F157-, R158-, L452R, T478K, D614G, P681R, D950N	64	3
Delta	T19R, T95I, E156G, F157-, R158-, L452R, T478K, D614G, P681R, D950N	31	4
Delta	T19R, T95I, G142D, Y145H, E156G, F157-, R158-, A222V, L452R, T478K, D614G, P681R, D950N	16	5
Delta	T19R, G142D, E156G, F157-, R158-, A222V, L452R, T478K, D614G, P681R, D950N	14	6
Delta	T19R, L452R, T478K, D614G, P681R, D950N	9	7
Delta	T19R, G142D, L452R, T478K, D614G, P681R, D950N	8	8
Delta	T19R, G142D, E156G, F157-, R158-, G181V, L452R, T478K, D614G, P681R, D950N	8	8
Delta	T19R, T95I, G142D, E156G, F157-, R158-, L452R, T478K, D614G, P681R, D950N, D1259Y	6	10
Delta	T19R, G142D, E156G, F157-, R158-, L452R, T478K, D614G, P681R, D950B	6	10
Delta	T19R, E156G, F157-, R158-, L452R, T478K, D614G, P681R	6	10
Delta	L5F, T19R, E156G, F157-, R158-, L452R, T478K, D614G, P681R, D950N	6	10
…	…	…	…
Omicron	A67V, H69-, V70-, T95I, G142D, V143-, Y144-, Y145-, N211-, L212I, G339D, S371L, S373P, S375F, K417N, N440K, G446S, S477N, T478K, E484A, Q493R, G496S, Q498R, N501Y, Y505H, T547K, D614G, H655Y, N679K, P681H, N764K, D796Y, N856K, Q954H, N969K, L981F	2	35

#### Functional impact score

While the Sequence Prevalence Score quantifies prevalence properties of variants, it requires some minimum time period to observe concerning trends and may not rank concerning variants high at early stages of emergence. A complementary approach for analyzing emerging variants independent of epidemiological dynamics is to predict the functional impact of amino acid substitutions using prior knowledge from experimental data. We have designated regions of the Spike protein that have been experimentally shown to impact immune escape, receptor binding affinity, or viral replication rates as “Sequence Features of Concern” (SFoC). We detail our procedure for selecting SFoCs in the following section, but overall, these SFoCs include sites that could impact the binding of one or more monoclonal antibody classes (Barnes et al., 2020), neutralization with infection- or vaccine- (e.g., mrna-1273)-induced antisera, receptor binding affinity, or other functionally important sites (e.g., NTD Supersite (McCallum et al., 2021), furin cleavage site (Liu et al., 2021; Escalera et al., 2022)).

**FIGURE 1:**
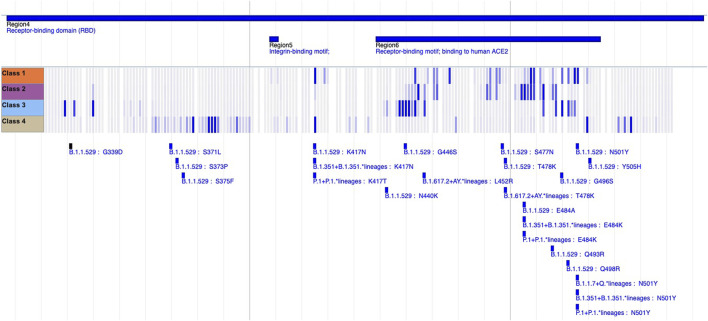
Sequence Features of Concern from Deep Mutational Scanning Monoclonal Antibody Escape Data. An image from the BV-BRC SARS-CoV-2 Variant Tracker (BV-BRC, 2022) genome browser shows a heatmap that quantifies the median escape fraction for each monoclonal antibody class (Class 1—4 tracks), with the darker blue indicating greater escape fraction, implying greater loss of antibody binding due to mutations at that site. We use these quantifications of potential antibody escape per mutated site per antibody class to define one type of Sequence Feature of Concern. By identifying overlap between variants (bottom tracks) and these monoclonal antibody escape Sequence Features of Concern, the Functional Impact Score of a covariant is computed.

Upon establishing our list of SFoCs, we assign each Spike variant a Functional Impact Score based on positional overlap with these important regions. For each mutated position in the variant, if an overlap is found with one of the regions in the SFoC list, a value of 1 is assigned, treating separately each of the monoclonal antibody classes, convalescent serum, vaccine serum, ACE2 binding affinity, NTD supersite, and furin cleavage site. Summing all the values of each position mutated in the variant produces the Functional Impact Score. Figure 1 provides a visual representation of this positional overlap with SFoCs defined by deep mutational scanning monoclonal antibody escape data using the genome browser of the BV-BRC SARS-CoV-2 Variant Tracker (BV-BRC, 2022).

#### Sequence features of concern

Most of the data used to define the SFoCs were derived from the deep mutational scanning experiments conducted by the Bloom Lab, which quantified the mutation impact towards monoclonal antibody escape, convalescent serum antibody escape, vaccine (mrna-1273)-elicited antibody escape, and ACE2 binding affinity for nearly every position in the Receptor Binding Domain (RBD) (Starr et al., 2020; Greaney et al., 2021a; Starr et al., 2021a; Greaney et al., 2021b; Starr et al., 2021b; Greaney et al., 2021c; Starr et al., 2021c; Dong et al., 2021; Tortorici et al., 2021). To achieve these quantifications, the Bloom Lab constructed an RBD mutant library such that each amino acid site on the RBD was mutated with the 19 possible amino acid substitutions in the genetic background of the Wuhan-Hu-1 reference strain. Rigorous statistical processing was used to calculate an “escape fraction” for antibody escape (between 0 and 1) and “binding average” for ACE2 affinity (between -5 and 1) for every mutation at every position of the RBD. Each monoclonal antibody belonging to one of the four Barnes et al. structural epitope classes (Barnes et al., 2020), subject specific convalescent sera, and subject specific vaccine sera had their own escape fraction scores per mutation per site. The data from all these deep mutational scanning studies can be downloaded and explored at https://jbloomlab.github.io/SARS2_RBD_Ab_escape_maps/.

The distribution of scores for each antibody escape datasets and the ACE2 binding dataset were examined to identify positions of the RBD showing significant increases in antibody escape or ACE2 binding affinity upon mutation. The analysis of antibody escape data showed that most of the escape fraction values were close to the 0 baseline and less than 0.2 (Supplementary Figure S1A). Therefore, to capture positions on the RBD that led to strong antibody escape when mutated, an escape fraction threshold of >0.25 was applied. Consequentially, the antibody escape SFoCs were defined as RBD sites with one or more mutations that lead to an escape fraction exceeding this threshold for some monoclonal antibody, convalescent subject sera, or vaccine subject sera. The monoclonal antibodies corresponding to a mutation exceeding this escape fraction threshold were categorized into their structural epitope class to generalize the scoring for functional impact. As a result, 75 sites on the RBD were designated as having a significant predicted impact on the binding of one or more of four antibody classes and 36 sites as having a significant impact on the binding of antibodies from convalescent or vaccine sera.

Similarly, we evaluated the distribution of scores for the ACE2 binding affinity dataset. This analysis showed that most scores were close to the 0 baseline or negative, where a negative score implied a decrease in binding affinity (Supplementary Figure S1B). To identify mutations that led to increased ACE2 binding affinity, a binding average threshold of >0.1 was selected, thus designating 12 sites that could significantly increase the binding to ACE2 upon mutation, including site 501, which leads to high degree of conformational alterations of the Spike RBD when bound to ACE2 when mutated (Gupta, 2022).

The remaining Sequence Features of Concern were those deemed critical for adaptive immunity or viral tropism, such as the NTD supersites (McCallum et al., 2021) (Spike positions 14–20, 140–158, 245–264), position 614 (Korber et al., 2020), and the region flanking the furin cleavage site (Liu et al., 2021; Escalera et al., 2022) (sites 671–692), determined through literature curation.

#### Composite score

The *Composite Score* is computed by summing the *Sequence Prevalence Score* and the *Functional Impact Score*. The Composite Score simultaneously identifies variants with alarming sequence prevalence dynamics AND variants that would be predicted to impact important functional characteristics of the virus. For example, although the initial analysis of the Omicron variant using sequence data from November 2021 did not show a high Sequence Prevalence Score (Table 1), the original Omicron sequence did show a very high Functional Impact Score and therefore produced a high Composite Score (Table 2). These results highlight the importance of both the Sequence Prevalence Score and Functional Impact Score for the early identification of Variants of Interest for further evaluation.

**TABLE 2:** Global Spike Covariant Ranking with the Composite Score. The output of a ranking based on GISAID data up to November 2021. The analysis returns a global ranking for all Spike variants based on the *Composite Score*. In this case, the Omicron variant jumped considerably in rank relative to the ranking based on the Sequence Prevalence Score alone as shown in Table 1, thus showing the impact of quantifying the Functional Impact Score in overall variant rankings.

WHO label	Covariant	Sequence prevalence score	Functional impact score	Composite score	Rank
Delta	T19R, G142D, E156G, F157-, R158-, L452R, T478K, D614G, P681R, D950N	123	10	133	1
Delta	T19R, T95I, G142D, E156G, F157-, R158-, L452R, T478K, D614G, P681R, D950N	92	10	102	2
Delta	T19R, E156G, F157-, R158-, L452R, T478K, D614G, P681R, D950N	64	9	73	3
Delta	T19R, T95I, E156G, F157-, R158-, L452R, T478K, D614G, P681R, D950N	31	9	40	4
Omicron	A67V, H69-, V70-, T95I, G142D, V143-, Y144-, Y145-, N211-, L212I, G339D, S371L, S373P, S375F, K417N, N440K, G446S, S477N, T478K, E484A, Q493R, G496S, Q498R, N501Y, Y505H, T547K, D614G, H655Y, N679K, P681H, N764K, D796Y, N856K, Q954H, N969K, L981F	2	36	38	5
Delta	T19R, T95I, G142D, Y145H, E156G, F157-, R158-, A222V, L452R, T478K, D614G, P681R, D950N	16	11	27	6
Delta	T19R, G142D, E156G, F157-, R158-, A222V, L452R, T478K, D614G, P681R, D950N	14	10	24	7
Delta	T19R, G142D, E156G, F157-, R158-, G181V, L452R, T478K, D614G, P681R, D950N	8	10	18	8
Delta	T19R, T95I, G142D, E156G, F157-, R158-, L452R, T478K, D614G, P681R, D950N, D1259Y	6	10	16	9
Delta	T19R, G142D, E156G, F157-, R158-, L452R, T478K, D614G, P681R, D950B	6	10	16	9
Delta	T19R, G142D, L452R, T478K, D614G, P681R, D950N	8	8	16	9
Delta	T19R, L452R, T478K, D614G, P681R, D950N	9	7	16	9
Delta	T19R, T95I, G142D, E156G, F157-, R158-, L452R, T478K, D614G, Q677H, P681R, T859I, D950N	4	11	15	13
Delta	T19R, G142D, E156G, F157-, R158-, L452R, T478K, D614G, P681R, D950N, G1167V	5	10	15	13
Delta	T19R, E156G, F157-, R158-, L452R, T478K, D614G, P681R	6	9	15	13

#### Single mutation and PANGO lineage scoring

In addition to the *Composite Score* and its counterparts, we devised methods that rely solely on epidemiological dynamics for scoring single amino acid mutations and PANGO Lineages, denoted as the *Mutation Prevalence Score* (Supplementary Table S1A,B) and the *Emerging Lineage Score* (Supplementary Table S2A,B), respectively. The details of these two methods are provided in the Supplementary Material. They rely on a similar algorithm used for the Sequence Prevalence Score. The Emerging Lineage Score algorithm differs slightly from the Sequence Prevalence Score; Supplementary Figure S2 provides context as to how the algorithm was devised. Ranking PANGO lineages with the Emerging Lineage Score can facilitate selection of lineages for conducting a specific Composite Scoring analysis of covariants within the specific PANGO lineage (Supplementary Table S3).

### Signals of early detection and virus evolution

This approach has been successful at catching signals of concerning variants as they emerged. By January 2022, the most dominant lineage of Omicron clade was BA.1, which included several sub-lineages with varying Spike covariants, most significantly the addition of the R346K mutation. When scoring distinct Omicron covariants, differences between BA.1 + R346K and the original BA.1 covariant could be observed through an increase in Functional Impact Score (Table 3). BA.1 + R346K was designated as PANGO Lineage BA.1.1 the following month and noted as a Variant of Interest. In addition, a covariant of the BA.2 lineage displayed a Sequence Prevalence Score of 1, whereas many other covariants of the dominant BA.1 lineage showed a Sequence Prevalence Score of 0, providing an early signal that the BA.2 covariant should be monitored and evaluated. Indeed, the early detection analysis conducted the following month (February 2022) showed that the BA.2 covariant jumped from a score of 1–13 (Table 3). This BA.2 covariant eventually went on to reach a remarkably high Sequence Prevalence Score of 250 by the end of May. Among the more recent BA.4/BA.5 lineages, an early warning for a BA.4 covariant with a Spike V3G substitution was detected as soon as April, when a Sequence Prevalence Score of 1 was computed. By May, the BA.4 + V3G covariant had jumped from a score of 1–15. A BA.5 covariant with the Spike T76I substitution was also detected in May with an initial Sequence Prevalence Score of 1. By the end of June, BA.4 + V3G jumped from 15 to 24 and BA.5 + T76I jumped from 1 to 8. The BA.4 + V3G covariant eventually surpassed all BA.2 covariants in Sequence Prevalence Score in July, and other BA.5 covariants, such as BA.5 with K440N reversion and BA.5 with S408R reversion, were showing increased signals with Sequence Prevalence Scores of 32 and 22, respectively. Finally, by end of August, BA.5 covariants had the most dominant Sequence Prevalence Scores. These examples show how signals of sequence prevalence and functional impact provided early evidence of emerging variant dynamics that were used by the SAVE consortium *in vitro* and *in vivo* groups for experimental prioritization.

**TABLE 3:** Global Spike Variant Ranking for a Selected Annotated Lists with the Composite Score. The output ranking from the Composite Score of selected covariants, annotated with names based on the addition or reversion of mutations relative to a PANGO lineage consensus variant constellation. **(A)** Using GISAID data up to January 2022, the scoring in the last three columns quantitatively capture how these covariants differ, including the increase in functional impact for BA.1 + R346K relative to the ancestral BA.1 and also captures an early signal for the BA.2 founder lineage. **(B)** Using GISAID data up to February 2022, these scorings capture the 13-fold increase in Sequence Prevalence Score relative to the January results for the same BA.2 founder lineage, strongly flagging an emerging Variant of Concern.

(A)	Sequence prevalence score	Functional impact score	Composite score
Covariant^a^			
B.1.617.2 + T95I	78	10	88
BA.1 (Omicron)	22	36	58
BA.1 + R346K	12	38	50
BA.1 + A701V	2	36	38
BA.1 + K417_ + N440_ + G446_	7	29	36
BA.1 + I1081V	0	36	36
BA.3	0	35	35
BA.2	1	34	35
BA.1 + R346K + K417_ + N440_ + G446_	3	31	34
BA.1 + K417_ + N440_ + G446_ + L452R + A701V	0	32	32
BA.1 + K417_ + N440_ + G446_ + L452R	0	32	32
BA.2 + K417_ + N440_	0	30	30
BA.1 + K417_ + N440_ + G446_+ I1081V	0	29	29
BA.3 + K417_ + N440_ + G446_	0	28	28

(B)	Sequence prevalence score	Functional impact score	Composite score
Covariant^a^			
BA.1	74	36	110
BA.1 + R346K	57	38	95
BA.1 + A701V	12	36	48
BA.2	13	34	47
BA.1 + K417_ + N440_ + G446_	15	29	44
BA.1 + F643L + A701V	6	36	42
BA.1 + K417_	8	33	41
BA.1 + N211_ + L212_ + 214_	3	36	39
BA.1 + I1081V	2	36	38
BA.2 + L24_ + P25_ + P26_ + A27_	2	34	36
BA.3	0	35	35
BA.1 + K417_ + N440_ + G446_ + L452R	0	32	32
BA.1 + N211_ + L212_ + 214_ + G339_ + S371_ + S373_ + S375_ + K417_ + N440_ + G446_ + L452R	0	30	30
B.1.617.2 + T95I	20	10	30

^a^ Underscore (_) = Ancestral reversion (no mutation at the site).

### Visualizing early detection

To complement the early detection analysis scoring algorithms, it has also been useful to visualize variant growth both globally and regionally to further understand the dynamics of these variants and facilitate early detection.

#### Visualizing relative growth of PANGO lineages

**FIGURE 2:**
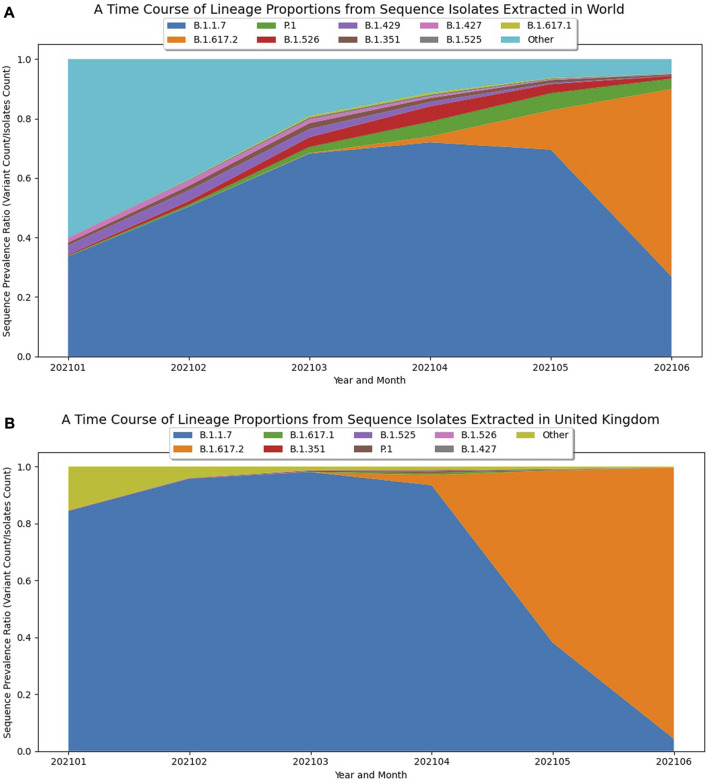
Visualizing the Emergence of the B.1.617.2 (Delta) Variant. **(A)** A plot of global trends over time based on GISAID data up to June 2021 to visualize the early growth dynamics of B.1.617.2 (Delta). The graph displays the 10 PANGO lineages with the most substantial global prevalence dynamics over a six-month time frame, with the early emergence of B.1.671.2 and the sudden sharp decline of B.1.1.7 (Alpha) clearly evident. **(B)** A plot of growth trends for Delta in the United Kingdom based on GISAID data up to June 2021. The graph displays the PANGO lineages with the most substantial global prevalence dynamics over a six-month time frame solely within the United Kingdom and shows the local growth of B.1.617.2 and decline of B.1.1.7.

When a new variant displays alarming epidemiological dynamics or predicted functional changes, like Delta and Omicron, researchers may want to visualize the growth dynamics of the new variant in the context of how the prevalence of other variants are changing. During viral evolution, when a new variant/strain becomes dominant, it triggers a phenomenon where the prevalence of the currently circulating variants suddenly begin to sharply decline, perhaps because the new variant has a fitness advantage and is able to outcompete the older variants (Korber et al., 2020; Martin et al., 2021; McCrone et al., 2021; Zhou et al., 2021). If the prevalence of a novel variant with alarming characteristics is increasing with a corresponding sudden decline in growth of other circulating variants, this would further indicate the early detection of a potential Variant of Concern. As an example, prior to the emergence of Delta, B.1.1.7-derived Alpha variants were the most dominantly circulating variants around the world. However, between May and June of 2021, it was becoming clear that the newly emerging Delta variant was displaying noteworthy properties (McCrone et al., 2021) and very quickly replaced the other circulating variants, including B.1.1.7 (Alpha), both globally (Figure 2A) and regionally, especially in the UK (Figure 2B). By visualizing these dynamics in stacked line plots, the relative magnitude by which a novel lineage is growing with respect to other lineages offers further evidence of the early detection of a potential Variant of Concern.

#### Visualizing growth of covariants

**FIGURE 3:**
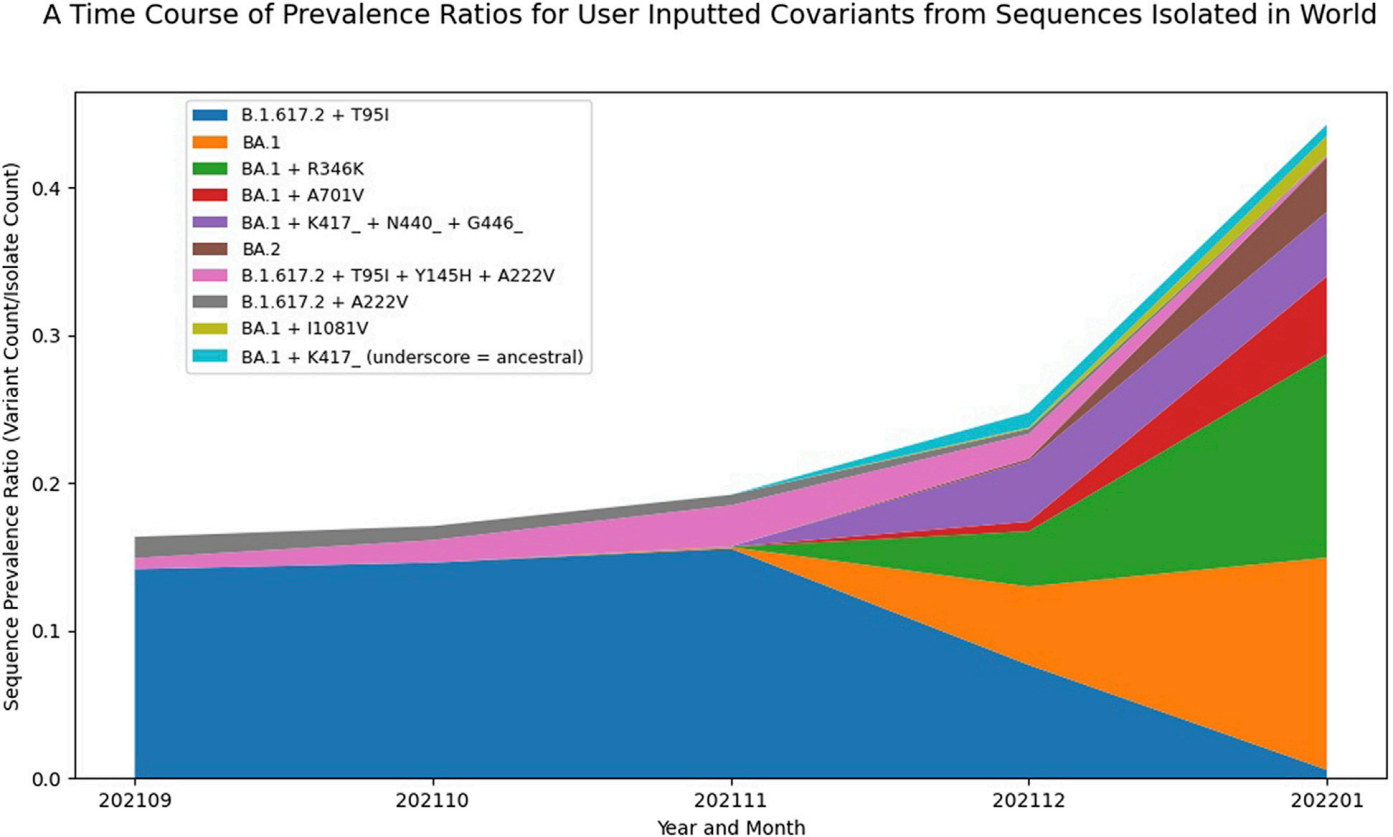
Visualizing the Emergence of Omicron Variant Constellations. A plot displaying the prevalence dynamics over six months of selected Spike covariants based on GISAID data up to January 2022. This visualization captures the sharp growth and relatively large global prevalence of BA.1 + R346K as well as the early growth of a BA.2 covariant. The names presented in the legend use the ancestral PANGO lineage designation plus or minus certain amino acid mutations to represent different covariants. Underscores indicate that certain mutations were reverted to ancestral relative to the parent PANGO lineage.

In addition to tracking the emergence and growth of new PANGO lineages, it is also useful to visualize the evolution and growth of variants within these lineages. As discussed earlier, the B.1.1.529 (Omicron) started to show rapid growth in December 2021 and then quickly accumulated additional substitutions, ultimately generating the BA.1 and BA.2 lineages and sub-lineages. By providing a list of covariants of interest, like the one used in Table 3, a graph showing the prevalence of the selected covariant list was produced (Figure 3). Comparing the Composite Score results from Table 3 with the visualization in Figure 3, some interesting insights about BA.1 + R346K emerged. BA.1 + R346K had a high functional impact score, and while the Sequence Prevalence Score was also high, it was not quite as high as BA.1 for the month of January 2022. However, Figure 3 shows that BA.1 + R346K was exhibiting a sharper change in its global prevalence relative to BA.1, suggesting that this variant would warrant further monitoring. Additional important insights for a BA.2 covariant also emerged from these data, showing that although the Sequence Prevalence Score was only one, it was coinciding with sharp relative growth globally in just a single month. Indeed, these plots provide a complementary representation to the Composite Score ranking to facilitate early detection analysis and more confidently identify variants that warrant experimental evaluation.

#### Visualizing growth of individual amino acid substitutions

**FIGURE 4:**
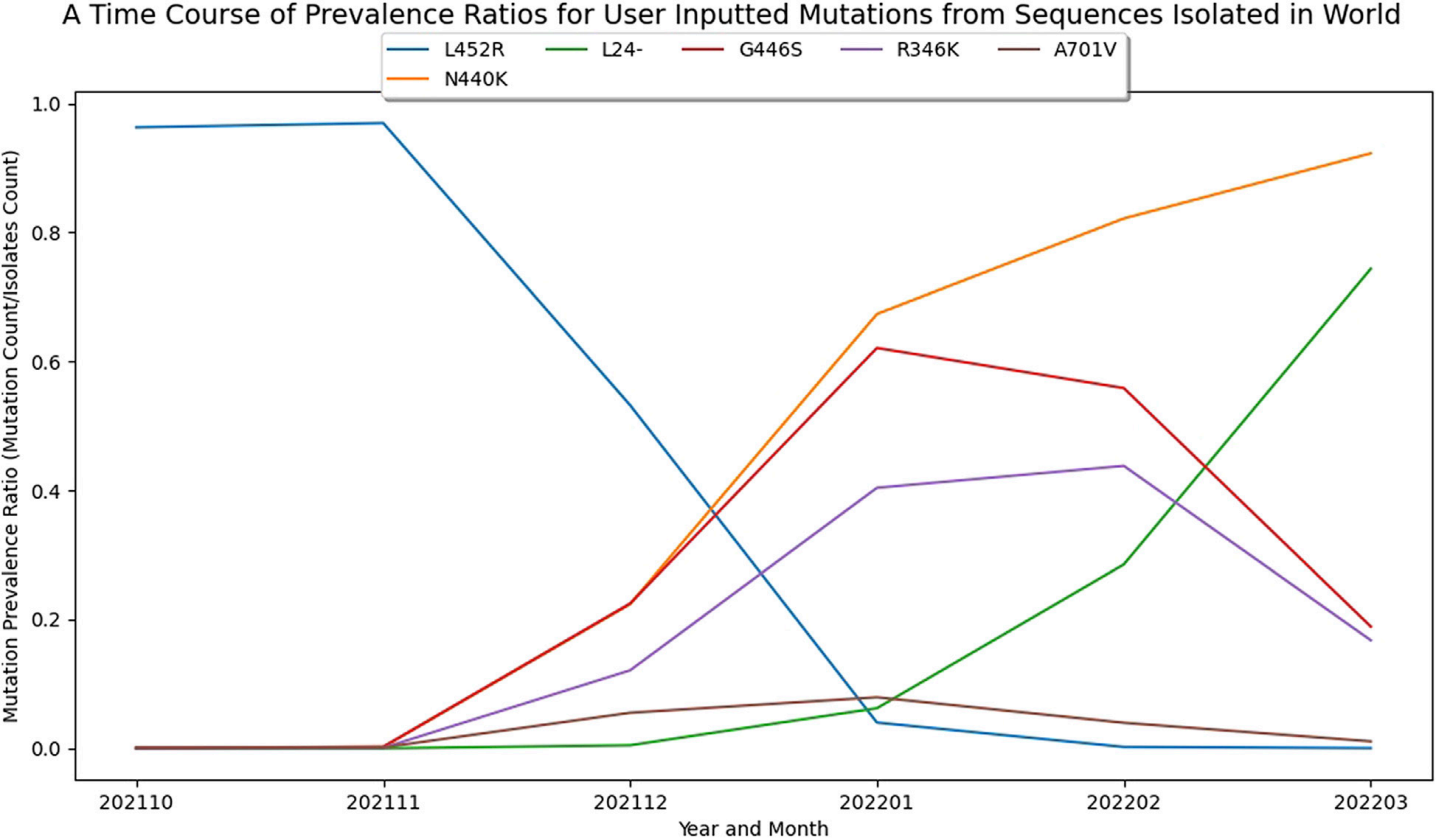
Visualizing Growth Patterns of Single Amino Acid Mutations in the Spike Protein. A plot based on GISAID data up to March 2022 demonstrating the shift in dynamics for individual amino acid mutations. This plot is based on six selected amino acid mutations. Note that the L24-is a part of an extended deletion that also includes P25 and P26 found in BA.2.

In addition to the analysis of lineages and covariants, analysis of individual amino acid mutations that contribute to multiple covariants/lineages, perhaps due to convergent evolution or recombination, are also worth exploring. For example, through March 2022, the L24-, R346K, N440K, G446S, L452R, A701V mutations were appearing sporadically throughout our ranked covariants. Plotting the dynamics of these individual amino acid mutations over subsequent time periods shows that the R346K and G446S mutations started to decrease in prevalence at the same time as the prevalence of L24-was rapidly increasing, suggesting that viruses carrying this mutation may possess a fitness advantage (Figure 4). Indeed, this L24-is part of an extended deletion that distinguishes BA.2, which subsequently replaced BA.1 in global dominance.

## Challenges

Defining SARS-CoV-2 variants that warrant functional experimental evaluation presents significant challenges. Early genomic sequencing data is often subject to data imbalances and ascertainment biases with respect to specific geographic regions. Wealthier countries with higher sequencing capacity, such as the US and UK, are responsible for most publicly deposited SARS-CoV-2 genome sequences. A survey of the 4.5 million SARS-CoV-2 genomes available in GenBank and BV-BRC through April 2022 showed that roughly 47% were from the US and 38% from the UK. Thus, the evaluation of variant dynamics is biased to the changes occurring in these geographic regions. Sequence ambiguity that can occur as a new variant emerges, before the sequencing assays can be optimized, presents an additional challenge. Since the SARS-CoV-2 genomes submitted to the public databases have already been assembled and a consensus sequence called, the quality of the read level data cannot be easily evaluated independently. For example, in the processing of variant data through the GISAID metadata file downloads, many sequences appeared to have reverted to ancestral residues in comparison to the original Omicron outbreak sequences. However, in many cases this was due to low sequence coverage in certain genomic regions that is not apparent in the metadata file. Indeed, processing assembled GenBank sequence data from BV-BRC, only about 25% of sequences had little to no ambiguities, and the amount of ambiguity in the sequencing data fluctuated during the initial emergence of certain important variants, like Delta and Omicron, making it challenging to compute true sequence prevalence of authentic covariants. Another challenge is choosing the amount of data that ought to be regularly downloaded for computing the early detection scoring heuristics. While focusing on the most recent data could potentially be used to identify concerning variants more swiftly, a potential drawback would be sequence biases resulting in minimal representation. On the other hand, longer temporal data is more comprehensive and accurate, but could delay identification of newly emerging variants of concern. In our pipeline, we allow the option to choose anywhere from the past 2—6 months of global sequencing data to evaluate, with a default of 3 as our best attempt to set a balance between early detection and unbiased, accurate results. Finally, the most enduring challenge is the fact that these data are very large and continuously growing, as new SARS-CoV-2 sequence data are being deposited by the thousands every day. Designing pipelines to carry out real-time genomics analysis for this amount of data is technically challenging, and our techniques on how best to manage, analyze, and scale will need to continuously adapt.

## Discussion

Since the initial declaration of the COVID-19 pandemic, it has been clear that SARS-CoV-2 will persist in our community and remain a public health issue for the indefinite future. However, if we, as a biomedical community, come together and maximize our resources to combat this virus, we can continuously minimize the threat it brings to our world and transition to a phase in which SARS-CoV-2 becomes an endemic infection with only modest effects on public health. A major factor that contributes to minimizing the threat brought by SARS-CoV-2, or any other emerging pathogen, is through genomic surveillance. The best approach for monitoring viral evolution to ensure that we maintain reliable therapeutics and accurate diagnostics is by routinely collecting and sequencing samples from infected individuals to acquire complete virus genome sequences. With the COVID-19 outbreak, the research and public health communities have truly excelled at this task, as we have now reached a point where millions of SARS-CoV-2 genome sequences have been deposited in public databases. That being said, all of these data are only as powerful as the computational resources used to manage and analyze them. Thus, the explosion in publicly available viral genomes also calls for the development of appropriate computational frameworks that can scale as the data grows to enable the timely identification and prioritization of emerging variants for experimental evaluation.

In this work, we present approaches to process SARS-CoV-2 genomic sequencing data and epidemiological metadata on a regular basis and apply scoring heuristics to prioritize variants based on their epidemiological dynamics and predicted functional characteristics by computing Sequence Prevalence, Functional Impact, and Composite Scores. The output of these approaches provides concise lists of ranked variant constellations (covariants), offering a straightforward approach for wet-lab scientists to immediately determine which combinations of mutations ought to be evaluated.

These methods were validated through the early detection of the original Omicron variant (B.1.1.529/BA.1) and its subsequent sub-lineages (BA.2, BA.4, BA.5). Indeed, a high-ranking Functional Impact Score highlighted the initial emergence of Omicron in November 2021 and early Sequence Prevalence Score signals tagged the BA.2, BA.4, and BA.5 lineages in which their early scores jumped considerably in the subsequent month. These observations demonstrate the importance of monitoring all variants that achieve some initial score to determine if they will show increases in subsequent months as an indicator of rapid geographical spread. In addition to early prioritization of novel variants like Omicron, this system makes it easy to evaluate the subtle differences among the multitude of covariants arising from a single parent lineage, like comparing the BA.1 + R346K covariant to the original BA.1 covariant. Finally, the use of data visualizations for variant growth either by PANGO lineage, covariant, or single amino acid substitution demonstrates how coupling rankings with visualizations can further ground our confidence in early detection of variants that warrant experimental evaluation.

While the initial focus of this work has been on Spike protein variants, as that was NIAID SAVE’s interest for evaluation, the framework also can score and rank proteome-wide SARS-CoV-2 variant constellations and single amino acid substitutions, which are provided in our publicly available pipeline. While the primary focus was the Spike protein due to its critical role in adaptive immunity and viral tropism, the community is beginning to take serious interest in mutations arising in non-structural proteins, particularly nsp3, nsp5, and RNA-dependent RNA Polymerase (nsp12), due to their importance in the SARS-CoV-2 replication cycle and antiviral drug targeting (Tchesnokov et al., 2020; Martinot et al., 2021; Szemiel et al., 2021; Jochmans et al., 2022; Zhou et al., 2022). We continue to pay close attention to the literature to monitor the science behind non-Spike protein regions that play key roles in replication or impact drug targeting, and are continually updating the Sequence Features of Concern to account for this new knowledge. Ultimately, this framework can provide Composite Scores for variant constellations specific to any SARS-CoV-2 protein.

The methods presented in this work could be extended for evaluating variants of other viral species if sufficient data is available. This approach requires enough genomic sequencing data, consistent spatiotemporal isolation metadata, a methodology to compute variants with respect to a reference or a consensus genome (Zhang et al., 2017), and sufficient prior knowledge through experimental data to define Sequence Features of Concern and predict functional impacts. Moreover, these algorithms could be extended to evaluate genomic surveillance data from zoonotic disease reservoirs, such as influenza virus in avian or swine species. Indeed, several projects already exist to collect viral genomic sequences from such reservoirs and warehouse these data in public databases (Anderson et al., 2021). Overall, the methodologies described here can play an important role in a complete public health ecosystem by utilizing genomic sequencing data to monitor viral evolution and remain steps ahead of SARS-CoV-2, or any other virus, and ultimately deter the next pandemic.

## Data Availability

Data from NCBI Genbank, BV-BRC, and GISAID were analyzed in this work. The SARS-CoV-2 Early Detection and Analysis Pipeline and original contributions in this work can be found online at https://github.com/zwallace425/SARS-CoV-2_Pipeline.
