# Supplementary material for: Early detection of emerging SARS-CoV-2 variants of interest for experimental evaluation
Source: Front Bioinform. 2022 Oct 24;2:1020189. doi: 10.3389/fbinf.2022.1020189 (PMC9638046; doi:10.3389/fbinf.2022.1020189)
Supplement: Supplementary file 1 [file DataSheet1.docx]

**Supplementary Material**

**Additional Scoring Methods**

In addition to our methods for scoring SARS-CoV-2 Spike protein variant constellations through the *Sequence Prevalence Score, Functional Impact Score,* and the *Composite Score* as described in the main text, we devised two additional approaches for scoring single amino acid mutations, the *Mutation Prevalence Score*, as well as entire PANGO Lineages, the *Emerging Lineage Score*. Both methods rely on a similar algorithm used for the *Sequence Prevalence Score* and are supplied as analysis options in the *BV-BRC SARS-CoV-2 Early Detection and Analysis Pipeline.*

*Mutation Prevalence Score* – In addition to the covariant constellation analysis, a *Mutation Prevalence Score* analyzing single amino acid substitutions on the SARS-CoV-2 Spike protein is also calculated. As with the *Sequence Prevalence Score*, this approach uses data from the past three months to assign a score of 1 for every country/month combination in which the prevalence of an amino acid mutation is >5% or the growth rate is greater than 5-fold. **Supplemental Table 1A-B** show the results of ranking mutations within the RBD and NTD using global GISAID sequence data from December 2021.

*Emerging Lineage Score* – In some cases, researchers may just want to know how the various PANGO Lineage designations differ in terms of their epidemiological dynamics. As we saw with Delta, there were a multitude of AY.* lineages for the entire WHO clade designated in a relatively short time frame, so ranking these lineages would be helpful to identify which should be prioritized for further analysis.

As with the *Sequence Prevalence Score*, the *Emerging Lineage Score* begins by filtering for covariants with an assigned country and with a variant count greater than 10 in the most recent month. From there, using the past three months of data, for each PANGO lineage, every unique covariant/country/month combination in which the growth rate is greater than 15 is assigned a score of 1; these values are summed to compute the *Emerging Lineage Score*. Since this algorithm is counting multiple distinct covariant comprising each lineage, it could be biased towards PANGO lineages with abundant covariants; hence, we used a higher growth rate threshold to capture the key covariants driving the overall growth of the lineage. A growth rate threshold of 15 was chosen as it results in a relatively stable list of PANGO lineages. Growth rate thresholds above 15 did not significantly affect the results of the *Emerging Lineage Score* ranking, as we consistently noticed a moderate elbow at around 15 when plotting growth rate threshold versus size of the returned list of ranked PANGO Lineages for multiple months of scoring (**Supplemental Figure 2)**. This growth rate is also appealing in that it is relatively high and therefore relatively stringent. **Supplemental Table 2A-B** shows the results from this method using GISAID data from December 2021 and January 2022 to rank lineages globally. BA.1 was the dominant Omicron lineage in December 2021 and was the lineage with the highest *Emerging Lineage Score* (**Supplemental Table 2A**). However, by January 2022 additional Omicron lineages were rapidly growing with the presence of BA.1.1 and BA.2 (**Supplemental Table 2B**). Since these lineages are made up of multiple covariants, we could take the results returned from the *Emerging Lineage Score* to decide which covariants within these lineages warranted further investigation by running a PANGO lineage specific *Composite Score* ranking to prioritize the covariants within a specific lineage, as shown in **Supplemental Table 3** with BA.2, which captured a single covariant of the lineage with the strongest dynamics as early as January 2022.

**Early Detection Pseudocode/Algorithms**

The following details the algorithms of the entire pipeline to score and rank variant constellations or single amino acid mutations.

1.) Analysis from FASTA data

Input: Wuhan-Hu-1 reference in FASTA, last four months of SARS-CoV-2 NT sequences in FASTA, an optional protein

Output: List of variant constellations in form “[protein]_H10K,[protein]_D100G, …” scored and ranked by the *Composite Score* or single amino acid mutations ranked by *Mutation Prevalence Score*

Step 1: Quality Control

1. Iterate through all sequences in the FASTA and filter out sequences with high ambiguous nucleotide content (default >0.01), missing or incorrect viral species name, low sequence length coverage (default <29400 nucleotides), missing region, or missing date

Step 2: Pairwise Alignment to Wuhan-Hu-1 Reference Genome

For each SARS-CoV-2 NT sequence …

1. Pairwise align to Wuhan-Hu-1 reference genome
2. Parse the alignment cigar string to extract positions of all insertions and deletions in the query sequence and store appropriately
3. Using the annotated reference sequence gene start and end coordinates (Nextstrain/ViPR) and the positions of insertions and deletions on the query sequence, compute the gene start and end coordinates relative to the query
4. Translate each gene start and end on the query into the gene polypeptide chain
5. Abort current sequence and skip to next if a translated query gene is frameshifted (frameshift 🡪 ((len(ref polypeptide) – len(query polypeptide)) mod 3) != 0)

For each query gene polypeptide chain with a mutation …

- 1. Pairwise align the query gene polypeptide chain to the corresponding reference gene polypeptide chain
  2. Parse the alignment cigar string to extract positions of substitutions, insertions, and deletions
  3. Covert ORF1a/ORF1ab positions to non-structural protein position
  4. Concatenate the protein name, reference amino acid, mutation position, and alternate amino acid as [PROTEIN]_[REF][POS][ALT]
  5. Concatenate each mutation into one variant constellation, either for a single protein or the entire proteome

Step 3: Store Variant Counts Across Space and Time

For each computed variant constellation

1. Parse out the collection date and collection region from the original record
2. Store a hash table of variant-to-region-to-date counts
3. Store a hash table of region-to-date sequence isolate counts
4. Parse out each single mutation in the constellation, store in a hash table of mutation-to-region-to-date counts

Step 4: Compute the Variant Dynamics

1. Convert the hash tables into a data frame, with columns for variants, regions, dates, and counts
2. Bin the dates into a period (week/2-week/month) and aggregate the counts per region (or globally if user specified)
3. Use the region-to-date sequence isolates counts to compute a variant prevalence ratio per period per region (region variant count in period p / regional sequence isolates count in period p)
4. Use the region-period prevalence ratios to compute a region-period growth rate per variant (regional prevalence ratio in period p / regional prevalence ratio in period p – 1)
5. OPTIONAL: Use the region-period growth rates to compute a region-period jerk rate per variant (regional growth rate in period p – regional growth rate in period p-1)
6. End up with an analyzable data frame in the following format, stored as a pickle in the data directory of the pipeline

| **Variant** | **Region** | **Date** | **Variant Count** | **Isolates Count** | **Prevalence** | **Growth** | **Jerk** |
| --- | --- | --- | --- | --- | --- | --- | --- |
| X | X | YYYY-MM-DD | X | X | X | X | X |

Step 5: Score Variants Based on Dynamics and/or Predicted Functional Impact

1. Filter out variants with a variant count less than 10 in the most recent period
2. Exclude all data beyond the most recent three periods (default three months)

For each variant …

1. Count the times the variant has prevalence ratio greater than 0.05 or growth rate greater than 5 in any region-period combination 🡪 *Sequence Prevalence Score/Mutation Prevalence Score*

Only for scoring a variant constellation …

1. Count the overlap with *Sequence Features of Concern*; i.e., Spike regions shown to experimentally impact class 1, 2, 3, or 4 mAbs neutralization, convalescent sera/Moderna Abs neutralization, ACE2 binding, overlap with the NTD supersite, overlap with other critical Spike sequence feature regions such as the Furin Cleavage site, or any non-Spike drug resistant sites/active sites/mutatgenesis sites 🡪 *Functional Impact Score*
2. Sum the *Sequence Prevalence Score* with the *Functional Impact Score* 🡪 *Composite Score*

Step 6: Rank Variants by Composite Score/Mutation Prevalence Score

1. Sort the variants by their *Composite Score/Mutation Prevalence Score*
2. Save the results to a file stored in results directory

2.) Analysis from GISAID Metadata

Input: GISAID metadata file, an optional protein

Output: List of variant constellations in form “[protein]_H10K,[protein]_D100G, …” scored and ranked by the *Composite Score* or single amino acid mutations ranked by *Mutation Prevalence Score* or PANGO Lineages ranked by *Emerging Lineage Score*

Same as Step 3 – Step 6 in the previous example. For PANGO Lineage *Emerging Lineage Score*, parse out PANGO Lineage from GISAID metadata instead of the variant constellation.

**(A)**

**
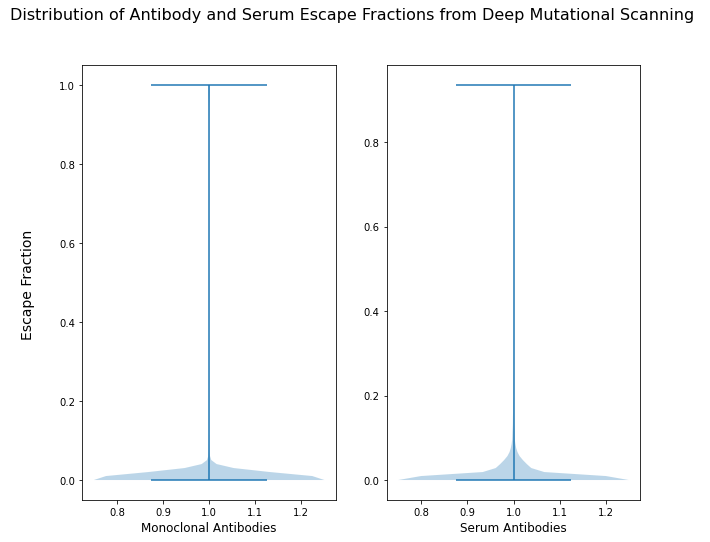
**

**(B)**


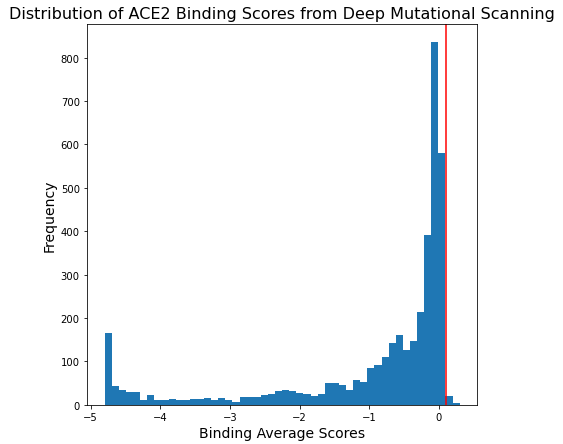


**Supplemental Figure 1 – Defining Sequence Features of Concern from a Threshold of Escape Fractions (A).** Violin plots of the distribution of escape fraction scores for monoclonal antibody escape and serum antibody escape from the Bloom Lab deep mutational scanning data. This distribution analysis led to an escape fraction threshold cutoff of 0.25 and subsequently led to the designation of 75 RBD sites significantly impacting monoclonal antibody binding and 36 sites impacting convalescent/Moderna vaccine (mrna-1273) sera elicited antisera binding upon mutation. **Defining Sequence Features of Concern from a Threshold of ACE2 Binding Scores (B).** The distribution of ACE2 binding average scores deep mutational scanning data. A score below 0 indicates a decrease in ACE2 binding affinity upon mutation, whereas a score above 0 indicates an increase in ACE2 affinity. Subsequently, this distribution analysis was used to select a threshold value of 0.1, that led to designating 12 RBD sites that could significantly increase ACE2 binding affinity upon mutation.


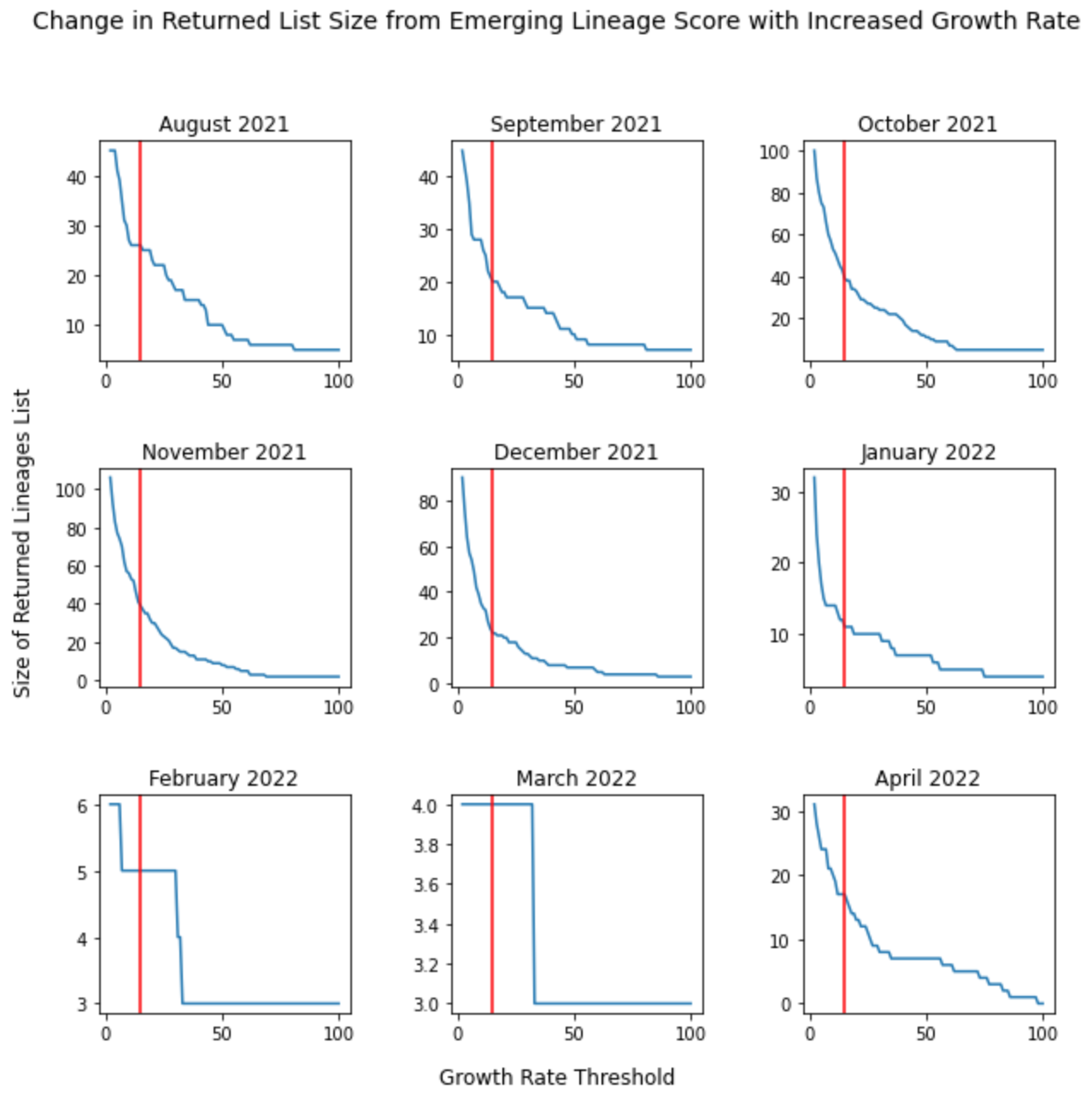


**Supplemental Figure 2: Defining a Growth Rate Threshold for the *Emerging Lineage Score***. Plots of growth rate threshold versus the returned list size from an *Emerging Lineage Score* for nine different months of analysis. Most of the plots per month demonstrate and elbow at around a growth rate of 15 (red vertical line), hence a month-to-month growth rate threshold of 15 was chosen for the *Emerging Lineage Score.*

**(A) (B)**

| **NTD Mutation** | **Mutation Prevalence Score** |
| --- | --- |
| G142D | 64 |
| T95I | 63 |
| T19R | 34 |
| F157- | 34 |
| R158- | 34 |
| E156G | 34 |
| Y144- | 30 |
| V143- | 30 |
| H69- | 29 |
| V70- | 29 |
| A67V | 28 |
| Y145- | 28 |
| N211- | 26 |
| L212I | 26 |
| T19I | 3 |
| A222V | 3 |
| Y145H | 3 |
| A27S | 3 |
| V213G | 2 |
| P25- | 1 |
| L24- | 1 |
| P26- | 1 |

| **RBD Mutation** | **Mutation Prevalence Score** |
| --- | --- |
| T478K | 64 |
| L452R | 34 |
| G339D | 30 |
| E484A | 26 |
| S477N | 26 |
| S373P | 25 |
| S375F | 25 |
| Q493R | 24 |
| N501Y | 23 |
| S371L | 20 |
| Q498R | 20 |
| G496S | 20 |
| K417N | 20 |
| Y505H | 19 |
| N440K | 18 |
| G446S | 18 |
| R346K | 15 |
| S371F | 2 |
| T376A | 2 |
| R408S | 2 |

**Supplemental Table 1 – Global Single Amino Acid Mutation Ranking with the *Mutation Prevalence Score***. **(A)** The output of an RBD mutation ranking and **(B)** the output of an NTD mutation ranking based on GISAID data up to December 2021. The results return a ranking for Spike protein amino acid mutations ranked by the *Mutation Prevalence Score* within their respective domain, RBD or NTD*.*

**(A) (B)**

| **WHO Label** | **PANGO Lineage** | **Emerging Lineage Score** |
| --- | --- | --- |
| Omicron | BA.1 | 57 |
| Delta | AY.43 | 10 |
| Delta | AY.122 | 9 |
| Delta | AY.4 | 8 |
| Delta | B.1.617.2 | 6 |
| Delta | AY.127 | 4 |
| Delta | AY.121 | 4 |
| Delta | AY.4.2 | 3 |
| Delta | AY.43.4 | 2 |
| Delta | AY.3 | 2 |
| Delta | AY.39.1.1 | 2 |
| Delta | AY.39.1 | 2 |
| Delta | AY.4.5 | 1 |
| Delta | AY.4.2.3 | 1 |
| Delta | AY.29 | 1 |
| Delta | AY.46.6 | 1 |
| Delta | AY.58 | 1 |
| Delta | AY.39 | 1 |
| Delta | AY.119 | 1 |
| Delta | AY.85 | 1 |
| Delta | AY.36 | 1 |
| Delta | AY.111 | 1 |

| **WHO Label** | **PANGO Lineage** | **Emerging Lineage Score** |
| --- | --- | --- |
| Omicron | BA.1 | 103 |
| Omicron | BA.1.1 | 46 |
| Omicron | BA.2 | 15 |
| Delta | AY.4 | 3 |
| Delta | AY.122 | 2 |
| Delta | AY.69 | 1 |
| Delta | AY.43 | 1 |
| Delta | AY.39.1.1 | 1 |
| Delta | B.1.617.2 | 1 |
| Delta | AY.102 | 1 |
| Delta | AY.132 | 1 |

**Supplemental Table 2 – PANGO Lineage Ranking with the *Emerging Lineage Score.*** The output of a lineage ranking based on GISAID data up to December 2021 **(A)** and January 2022 **(B)**. The results return a global ranking of PANGO Lineages based on the *Emerging Lineage Score.* In December 2021, BA.1 was the strongest emerging lineage with other Delta sub-lineages still on the rise. However, by January 2022, several other Omicron lineages were also emerging, with the Delta lineages tapering off.

| **Covariant** | **Sequence Prevalence Score** | **Functional Impact Score** | **Composite Score** |
| --- | --- | --- | --- |
| T19I, L24-, P25-, P26-, A27S, G142D, V213G, G339D, S371F, S373P, S375F, T376A, D405N, R408S, K417N, N440K, S477N, T478K, E484A, Q493R, Q498R, N501Y, Y505H, D614G, H655Y, N679K, P681H, N764K, D796Y, Q954H, N969K | 13 | 34 | 47 |
| T19I, G142D, V213G, G339D, S371F, S373P, S375F, T376A, D405N, R408S, K417N, N440K, S477N, T478K, E484A, Q493R, Q498R, N501Y, Y505H, D614G, H655Y, N679K, P681H, N764K, D796Y, Q954H, N969K | 2 | 34 | 36 |
| T19I, L24-, P25-, P26-, A27S, G142D, G339D, S371F, S373P, S375F, T376A, D405N, R408S, K417N, N440K, S477N, T478K, E484A, Q493R, Q498R, N501Y, Y505H, D614G, H655Y, N679K, P681H, N764K, D796Y, Q954H, N969K | 2 | 34 | 36 |
| T19I, L24-, P25-, P26-, A27S, G142D, V213G, G339D, S371F, S373P, S375F, T376A, D405N, R408S, K417N, S477N, T478K, E484A, Q493R, Q498R, N501Y, Y505H, D614G, H655Y, N679K, P681H, N764K, D796Y, Q954H, N969K | 2 | 33 | 35 |
| T19I, L24-, P25-, P26-, A27S, G142D, V213G, G339D, S371F, S373P, S375F, T376A, D405N, R408S, K417N, N440K, S477N, T478K, E484A, Q493R, Q498R, N501Y, Y505H, D614G, H655Y, N679K, P681H, N764K, D796Y, G798D, Q954H, N969K | 1 | 34 | 35 |
| T19I, G142D, V213G, G339D, S371F, S373P, S375F, T376A, D405N, R408S, K417N, S477N, T478K, E484A, Q493R, Q498R, N501Y, Y505H, D614G, H655Y, N679K, P681H, N764K, D796Y, Q954H, N969K | 2 | 33 | 35 |
| T19I, L24-, P25-, P26-, A27S, V213G, G339D, S371F, S373P, S375F, T376A, D405N, R408S, K417N, N440K, S477N, T478K, E484A, Q493R, Q498R, N501Y, Y505H, D614G, H655Y, N679K, P681H, N764K, D796Y, Q954H, N969K | 1 | 33 | 34 |
| T19I, L24-, P25-, P26-, A27S, V213G, G339D, S371F, S373P, S375F, T376A, D405N, R408S, K417N, S477N, T478K, E484A, Q493R, Q498R, N501Y, Y505H, D614G, H655Y, N679K, P681H, N764K, D796Y, Q954H, N969K | 1 | 32 | 33 |
| T19I, L24-, P25-, P26-, A27S, G142D, V213G, G339D, S371F, S373P, S375F, T376A, D405N, R408S, K417N, N440K, E484A, Q493R, Q498R, N501Y, Y505H, D614G, H655Y, N679K, P681H, N764K, D796Y, Q954H, N969K | 1 | 32 | 33 |
| T19I, L24-, P25-, P26-, A27S, G142D, V213G, G339D, S371F, S373P, S375F, T376A, D405N, R408S, N440K, S477N, T478K, E484A, Q493R, Q498R, N501Y, Y505H, D614G, H655Y, N679K, P681H, N764K, D796Y, Q954H, N969K | 1 | 31 | 32 |

**Supplemental Table 3 – Global Ranking of BA.2 Variants with the *Composite Score***. The output of a *Composite Score* ranking based on GISAID data up to January 2022. The purpose of this ranking is to focus the analysis on covariants within a specific lineage, in this case within BA.2. The ability to capture a dominant covariant (top row) likely driving much of the observed dynamics for this lineage can be observed.
